# The immediate risk of cancer and its precursor lesions in women with abnormal cervical glandular cytology

**DOI:** 10.7150/jca.99757

**Published:** 2025-01-01

**Authors:** Yang Li, Yao Chen, Wanrun Lin, Keyi Chen, Weiguo Lv, Feng Zhou

**Affiliations:** 1 Department of Gynecologic Oncology, Zhejiang University School of Medicine Women's Hospital, Hangzhou, Zhejiang Province, China, 310006.; 2 Zhejiang Key Laboratory of Maternal and Infant Health, China, 310006.; 3 Department of Obstetrics and Gynecology, The First Affiliated Hospital of Ningbo University, Ningbo, Zhejiang Province, China, 315010.; 4 Laboratory of Pathology, National Cancer Institute, National Institutes of Health, Bethesda, MD, USA, 20892.; 5 Zhejiang Provincial Clinical Research Center for Obstetrics and Gynecology, China, 310006.; 6 The International Peace Maternal and Child Health Hospital, School of Medicine, Shanghai Jiao Tong University, Shanghai, 200030.; 7 Shanghai Key Laboratory of Embryo Original Diseases, Shanghai, China, 200030.

**Keywords:** abnormal cervical glandular cytology, hrHPV, risk stratification, CIN3+

## Abstract

**Objective**: This study aims to assess the immediate risk of cervical intraepithelial neoplasia grade (CIN)3+ lesions in women with abnormal cervical glandular cytology.

**Methods**: A total of 403 women with abnormal cervical glandular cytology who underwent simultaneous HPV genotyping and cervical biopsy at the Zhejiang University School of Medicine Women's Hospital, China, between 2016 and 2020, were included in this study. The probability of CIN3+ lesions among women in each group was further analyzed.

**Results**: Subsequently, 26.8% of women with abnormal cervical glandular cytology were diagnosed with CIN3+ lesions. The immediate risk of CIN3+ lesions in the atypical glandular cells, not otherwise specified (AGC-NOS), AGC-favor neoplasia (AGC-N), adenocarcinoma *in situ* (AIS), and adenocarcinoma (AC) groups were 12.7%, 55.7%, 88.9%, and 92.0%, respectively. The immediate risk of CIN3+ lesions in the AGC-NOS group was significantly lower than in any other groups. The positive rates of hrHPV in the AGC-NOS, AGC-N, AIS, and AC groups were 26.4%, 68.6%, 66.7%, and 56.0%, respectively. The prevalence of CIN3+ in the HPV-16 or 18/45 positive group was significantly higher than in the group of other 11 types positive and hrHPV negative group. Notably, women under 30 years old with AGC-NOS had a low risk of CIN3+ lesions (2.4%). When considering HPV status, the immediate risk of CIN3+ lesions in HPV-negative women was 0.0%.

**Conclusion**: hrHPV genotype and age are valuable indicators to assess the risk of CIN3+ in women with abnormal cervical glandular cytology. Women under 30 years old with AGC-NOS/HPV-negative may have the opportunity to delay colposcopy if appropriate.

## Introduction

Cervical cancer is the most prevalent malignant tumor of the female reproductive system, responsible for approximately 270,000 female deaths worldwide annually 1. The widespread implementation of Pap smear screening has significantly reduced the incidence and mortality of cervical cancer 2. High-risk human papillomavirus (hrHPV) plays a crucial role in the development of cervical precancerous lesions and cancers 3, 4. Many countries have adopted DNA-based screening tools like Hybrid Capture 2 assay (HC2) HPV and Cobas 4800 HPV tests as the primary screening method for women aged 25 years or older, leading to improved sensitivity 5-7.

However, unlike cervical squamous cell carcinoma (SCC), where almost all cases are linked to HPV infection, approximately 10% of endocervical adenocarcinomas (ECAs) in the general population and up to 25% in the Asian population are not associated with HPV 8-12. HPV negativity may result from the inclusion of endometrial cancers with secondary cervical involvement or metastatic tumors to the cervix. Notably, glandular epithelium does not facilitate productive HPV infection, leading to low accumulation of replicated episomal HPV DNA in infected cells, and only a limited number of HPV DNA copies are integrated into the cell genome 13. Consequently, such patients could benefit from combined cervical cytology and HPV testing 14.

Atypical adenocarcinoma (AGC) refers to a cytological abnormality diagnosed when there the pap smear shows abnormal cervical glandular cytology but lacks the characteristics of cervical adenocarcinoma *in situ* or invasive adenocarcinoma. A lot of studies have investigated the immediate histological results of women diagnosed with AGC, including a wide range of reactive changes, from mild inflammatory changes and glandular or squamous origin of pre-cervical cancer to invasive cervical cancer and other gynecologic cancers, such as endometrial cancer, ovarian cancer, fallopian tube cancer, etc, suggesting that women with AGC may exist multiple types of lesions.

In 2019, the American Society for Colposcopy and Cervical Pathology (ASCCP) updated the threshold for cervical intraepithelial neoplasia grade (CIN)3+ risk and the corresponding management strategies 15. As per the guidelines, women with an immediate CIN3+ risk of less than 4.0% were recommended to undergo follow-up surveillance, and their clinical management was guided by the 5-year risks of CIN3+ 15. However, there is a paucity of studies focusing on the immediate risk and follow-up management strategies for precancerous lesions in women with abnormal cervical glandular cytology. Therefore, the objective of this study is to elucidate the risk stratification of cancer and its precursor lesions in patients with abnormal cervical glandular cytology, based on HPV genotyping and age stratification.

## Materials and methods

### Patients and samples

The study was conducted with approval from the institutional review board at Women's hospital, School of medicine, Zhejiang university, China. We retrospectively reviewed 189,373 patients who had undergone the Aptima human papillomavirus (AHPV) assay and Pap smear test between September 2016 and May 2020 in the Zhejiang University School of Medicine Women's Hospital. The inclusive criteria include: 1) Women who visited our hospital between September 2016 and May 2020. 2) Women who underwent the AHPV assay and Pap smear test simultaneously. 3) The result of Liquid-Based Cytology shows glandular cell abnormalities and had follow-up biopsy and/or curettage during colposcopy examination during the next 6 months based on the ASCCP guidelines 15, 16. The exclusive criteria include:1) LBC shows the other results except the glandular cell abnormalities, such as negative for intraepithelial lesion or Malignancy (NILM) or low-grade squamous intraepithelial lesion (LSIL). 2) Women who had not underwent the AHPV assay at the same time. 3) Women who had not underwent the colposcopy examination within the next 6 months. Finally, a total of 403 cases were included in this study. The women finally enrolled were 19-85 years old (mean age 42.3 ± 11.1 years). Colposcopists were made aware of the cytology and AHPV results before the colposcopy visit was performed. The lack of histologic follow-up results of the rest of the patients is due to the patients being lost to follow up or receiving care elsewhere. This study was approved by the Ethics Committee of Zhejiang University School of Medicine Women's Hospital.

### Liquid-Based Cytology (LBC) and hrHPV mRNA testing

ThinPrep Pap tests (Hologic, Inc., San Diego, CA) were prepared following the manufacturer's instructions, and the slides were stained on a Sakura Tissue-Tek Automated Slide Stainer (Sakura Finteck USA, Torrance, CA). All cytology slides were diagnosed by cytopathologists in our hospital according to the 2014 Bethesda system 17. The LBC slides were classified into the following categories for the glandular cell abnormalities: 1) atypical glandular cells, not otherwise specified (AGC-NOS); 2) AGC-favor neoplasia (AGC-N); adenocarcinoma *in situ* (AIS); adenocarcinoma (AC). AHPV assay (Hologic, Inc., San Diego, CA) was performed with residual LBC samples following the manufacturer's instructions. The E6/E7 oncogenic mRNAs of 14 hrHPV genotypes (16, 18, 31, 33, 35, 39, 45, 51, 52, 56, 58, 59, 66, and 68) were tested by the Aptima HPV. HPV 16, or 18/45 genotype was further indicated in all AHPV-positive samples.

### Follow-up histopathologic diagnoses in patients

The cases with immediate histological correlation results including cervical biopsy and endocervical curettage performed within 6 months of the cytology and hrHPV co-testing were identified. The histopathologic results were categorized into the following general groups: (1) benign, (2) CIN1, (3) CIN2/3, (4) squamous cell carcinoma (SCC), (5) adenocarcinoma *in situ* (AIS), (6) adenocarcinoma (ADC), (7) adenosquamous carcinoma (ASC), (8) small cell carcinoma (SmCC), (9) non-cervical carcinoma. CIN3+, defined as CIN3 and worse. In patients with more than one tissue sample, the most abnormal diagnosis was recorded.

### Statistical analysis

The database was established by Excel and the data were statistically analyzed by SPSS 24.0 software (IBM, Armonk, NY, USA). Pearson chi- square or Fisher exact test was used to compare the distribution of cervical precancers and cancers in different hrHPV types and age groups. A p<0.05 was considered statistically significant.

## Results

### The prevalence of cervical precancers and cancers per HPV infection among women with abnormal cervical glandular cytology

**Figure 1:**
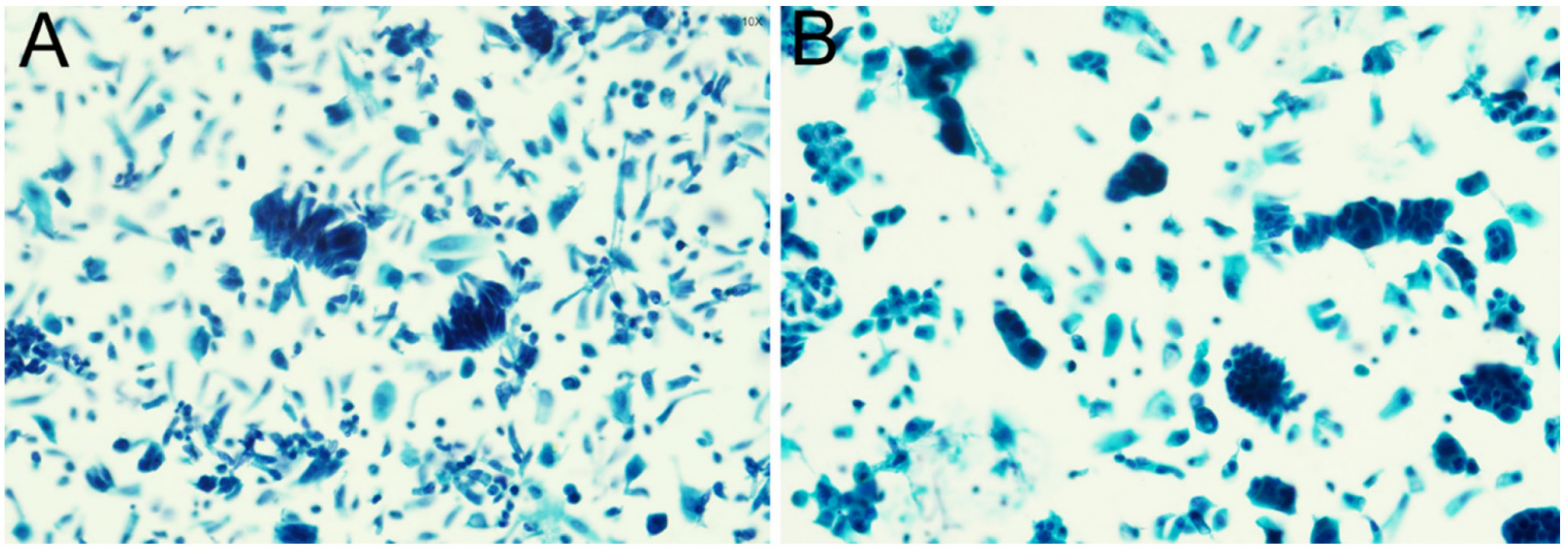
The representative images of abnormal glandular cells confirmed by the follow-up histologic examinations: usual type endocervical adenocarcinoma (A); gastric-type endocervical adenocarcinoma (B).

**Figure 2:**
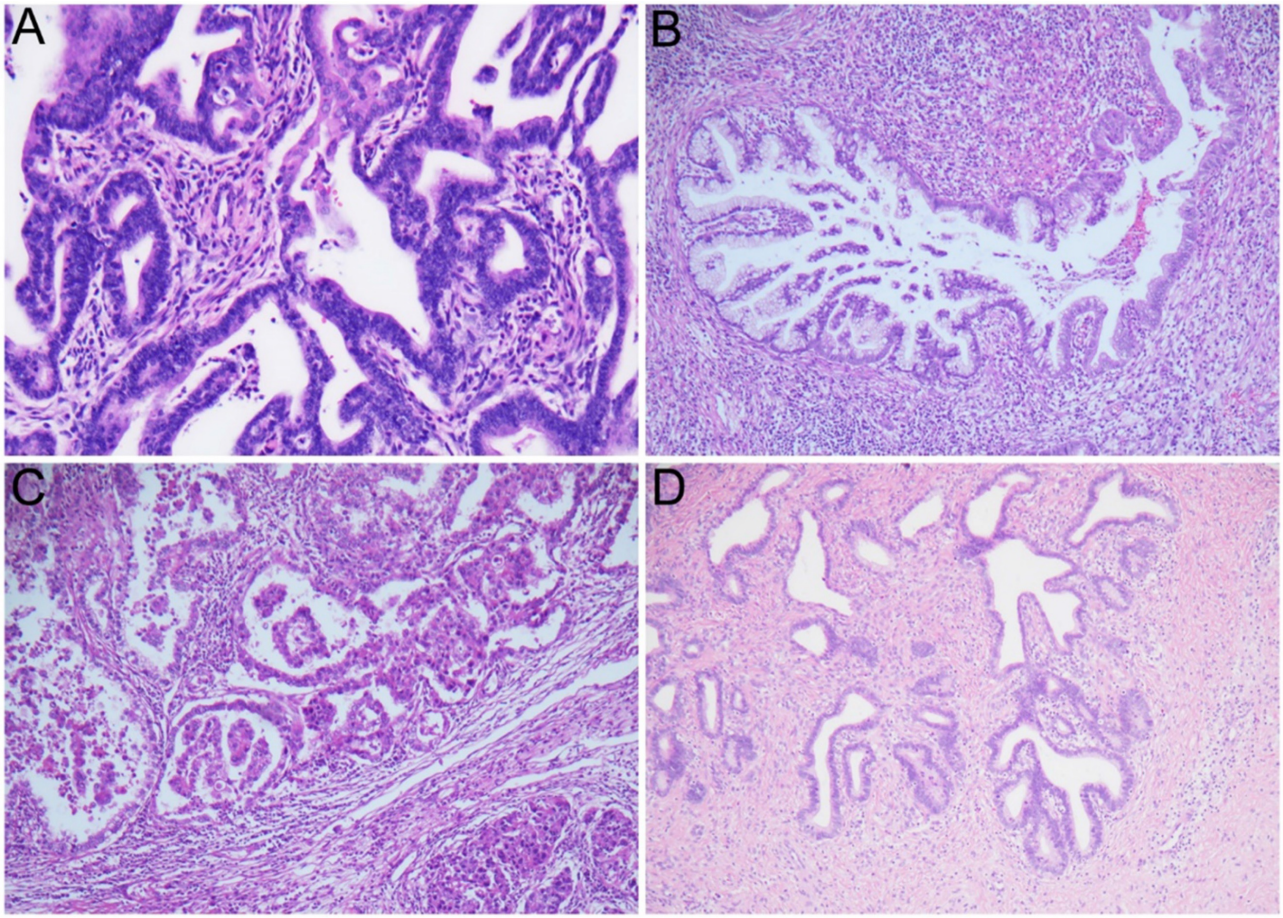
The representative images of usual type endocervical adenocarcinoma (A); gastric-type endocervical adenocarcinoma (B); clear cell adenocarcinoma (C); endocervical adenocarcinoma *in situ* (D).

A total of 403 cases with glandular cell abnormalities, hrHPV results, and immediate histologic results were included in this study, as shown in Table 1, 2. The mean age of all patients was 42.3 years (range 19 to 85). Among them, 299 (74.2%) were AGC-NOS, 70 (17.4%) were AGC-N, 9 (2.2%) were AIS, and 25 (6.2%) were AC. 256 (63.5%) women were hrHPV negativity and 147 (36.5%) women were hrHPV positivity. Among 147 hrHPV positive cases, 45 (11.2%) are HPV-16, 43 (10.7%) are HPV-18/45 and 58 (14.4%) are other 11 types of hrHPV positivity. Moreover, 1 (0.2%) woman showed HPV-16 and HPV-18/45 positivity. Follow-up histologic examinations revealed that 222 (55.1%) were regarded as benign, 62 (15.4%) were CIN1, 11 (2.7%) were CIN2, 23 (5.7%) were CIN3, 23 (5.7%) were AIS (including 2 gastric-type AIS), and 52 (12.9%) were cervical carcinoma including 43 ADC [30 usual type, 9 gastric-type, 4 mucinous type, 3 clear cell carcinoma (CCC)], 6 SCC, 2 ASC, and 1 SmCC. In addition, there are 10 cases of non-cervical carcinoma (2.5%), including 5 uterine origin, 2 ovarian origin, 3 gastrointestinal origin. The representative cytological and follow-up histologic images were showed in Figure 1, 2.

The immediate risk of cervical cancer and precancer in women with abnormal cervical glandular abnormalities was analyzed. As shown in Table 3, the immediate risk of CIN3+ lesions in AGC-NOS, AGC-N, AIS and AC groups were 12.7% (95% CI: 9.3-17.2), 55.7% (95% CI: 43.4-67.4), 88.9% (95% CI: 50.7-99.4) and 92.0% (95% CI: 72.5-98.6), respectively. The immediate risk of CIN3+ lesions in women in AGC-NOS group was significantly lower than any other groups (P<0.001). The relative risk of CIN3+ lesions in AGC-N, AIS and AC groups were 2.0 (95% CI: 1.51-2.57), 7.9 (95% CI: 1.28-49.89) and 10.9 (95% CI: 2.89-41.26) when compared with AGC-NOS group.

### HPV genotyping-stratified immediate histological correlation results among women with abnormal cervical glandular cytology

As shown in Table 4, the positive rates of hrHPV in AGC-NOS, AGC-N, AIS and AC groups were 26.4%, 68.6%, 66.7% and 56.0%, respectively. The positive rate of hrHPV in AGC-NOS group was significantly lower compared with the other three groups (P<0.001). However, there was no significant difference in the positive rate of hrHPV among AGC-N, AIS and AC groups. When further analyzed along the genotypes among patients with CIN3+ lesions: 62.2% (95% CI: 46.5-75.8) were HPV-16 positivity, 72.1% (95% CI: 56.1-84.2) were 18/45 positivity, 25.9% (95% CI: 15.7-39.3) were other 11 types of hrHPV positivity, 13.3% (95% CI: 9.5-18.2) were hrHPV negativity. Overall, 26.8% (108/403) were diagnosed with CIN3+ lesions. HPV-16 accounted for 25.9% (28/108), HPV-18/45 accounted for 28.7% (31/108), other 11 types of hrHPV accounted for 13.9% (15/108), and hrHPV negativity accounted for 31.5% (34/108). The prevalence of CIN3+ in HPV-16 or 18/45 positive group is significantly higher than that of other 11 types positive (P<0.001 and P<0.001 respectively) and hrHPV negative group (P<0.001 and P<0.001 respectively). There is no significant difference between group of other 11 types positive and hrHPV negative group. Overall, the prevalence of CIN3+ in hrHPV positive group is significantly higher than of hrHPV negative group (P<0.001). No statistical analysis was performed in the HPV-16 and HPV-18/45 dual positive group of due to limited case numbers (n=1).

### Age-stratified immediate histological correlation results among women with abnormal cervical glandular cytology

We further analyzed the distribution of histopathological examination results in different age groups. Table 5 showed the CIN3+ lesion prevalence among different age groups: 13.2% (7/53) were younger than 30 years, 23.1% (27/117) were aged 30-39 years, 31.5% (41/130) were aged 40-49 years, 32.0% (33/103) were aged over 50 years old. The majority (86.8%) of women with cervical glandular abnormalities were 30 years older. It showed that the immediate risk of CIN3+ was 13.2% (95% CI: 5.92-25.96) in women <30 years old, 23.1% (95% CI: 16.01-31.96) in women 30-39 years old, 31.5% (95% CI: 23.83-40.36) in women 40-49 years old, and 31.5% (95% CI: 23.83-40.36) in women older than 50 years. The immediate risk of CIN3+ was 13.2% in women <30 years old is lower than 40-49 and ≥50 years old age group (P=0.011 and P=0.011). The RR of women ≥50 years is the highest of 1.3 (95% CI: 1.08-1.51) when compared with <30 years old group. Generally, the probability of high-grade lesions increased with age.

### Immediate risk assessment of AGC-NOS women based on age and HPV status

As shown in Table 6, we further analyzed the immediate risk in AGC-NOS women with different age and hrHPV status. Overall, 12.7% (38/299) of women with AGC-NOS who were diagnosed with CIN3+ lesions were infected with hrHPV. Among them, women positive with HPV-16, HPV-18/45, other 11 types of hrHPV, and hrHPV negativity accounted for 23.7%, 21.1%, 23.7%, and 31.6%, respectively. The results showed that the majority (84.1%) of cases who were diagnosed as CIN3+ were 30 years older, only 1 case (1/38, 2.6%) was younger than 30 years. Moreover, we found hrHPV positivity and age ≥30 years were the risk factors in predicting the CIN3+ lesions, HPV-16 or 18/45 positive and age 40-49 years (both 50%) had the greatest impact on CIN3+ lesions. When taking 30 years as cut-off, it showed that the immediate risk of CIN3+ was 2.4% (95% CI: 0.1-14.4%) in women <30 years old and 14.3% (95% CI: 10.4%-19.4%) in women older than 30 years, with significant difference (P=0.034). AGC-NOS women with HPV negative and <30 years old may have the lowest immediate risk of CIN3+ lesions 0.0% (0/35). This proved that HPV status and age were covariant factors affecting the occurrence of high-grade lesions in women with AGC-NOS, which supported its role in the management of patients with AGC-NOS and could provide a basis for clinical triage.

## Discussion

It is widely acknowledged that the development of most cervical cancers and their precursors is contingent upon persistent HPV infection. Nevertheless, certain cervical primary carcinomas or metastatic carcinomas may not be HPV-dependent. Therefore, a combined test of cervical cytology and HPV might be more suitable for women in Asian countries. In our study, AGC-NOS/AGC-N cases constituted 91.6% of the total, while AIS/AC cases accounted for 8.4%. Among them, 63.5% of cases were hrHPV-negative, and 36.5% were hrHPV-positive. Subsequent histologic examinations revealed 5.7% as CIN3, 5.7% as AIS, 12.9% as cervical carcinomas, and 2.5% as non-cervical carcinomas. In a study by Pradhan *et al.* 18, histologic diagnoses of 3,709 AGC cases included negative (70.5%), low-grade squamous intraepithelial lesion (LSIL) and high-grade squamous intraepithelial lesion (HSIL) (20.7%), endocervical AIS/ADC (1.9%), and non-cervical carcinomas (6.0%).

Previous studies have reported that the incidence of AGC in cervical cytology ranges from 0.1% to 2.1% 19, and the incidence of AGC-N in AGC was found to be between 14.7% and 34.5% 20. In our study, the incidence of abnormal cervical glandular cytology was 0.2%, and AGC accounted for 0.19%, aligning closely with previous reports. Furthermore, we observed that up to 26.8% of cases with abnormal cervical glandular cytology exhibited CIN3+ lesions, with approximately 55.7% in the AGC-N group and 12.7% in the AGC-NOS group. The immediate risk of CIN3+ lesions in women with AGC-NOS was lower than in the AGC-N, AIS, and AC groups. A small-scale study in Thailand also retrospectively assessed the difference in the incidence of CIN3+ lesions between AGC-NOS and AGC-N groups, reporting a significant difference (P=0.02) 21. Shoji *et al.* also found a higher detection rate of malignant lesions in the AGC-N group compared to the AGC-NOS group 22.

Regarding age, Cheng *et al.* found that AGC women aged 60 and above were more likely to be diagnosed with cancer compared to those younger than 35 years old. This finding was supported by Aitken, who reported a higher proportion of immediate histological malignancies in AGC women over 50 years old 23, 24. Jin *et al.* divided all AGC patients into two groups, with a cutoff age of 50, and found that the incidence of CIN2+ lesions in women younger than 50 years old was significantly lower than in women older than 50 years 25. These findings were consistent with our study, as the majority (86.8%) of women with abnormal cervical glandular cytology were aged 30 years or older, especially those aged 50 years and above, supporting previous reports. Generally, older women with cervical glandular abnormalities, especially postmenopausal women, had a higher probability of high-grade lesions.

The hrHPV infection rate in women with AGC has been reported to be between 9.0% and 34.0%, and the hrHPV test can enhance the predictive ability of high-grade lesions in women with AGC 26-28. In our study, the prevalence of CIN3+ in the HPV-16 or 18/45 positive group was significantly higher than in the other 11 types positive and hrHPV nagative group. Similarly, a study from a large KPNC database demonstrated that the risk of malignancies was much lower in HPV-negative individuals than in HPV-positive individuals (0.37% vs. 9.0%) 29. The combined standardized cytological screening schedule and hrHPV test could facilitate the early diagnosis of high-grade lesions 28. A meta-analysis revealed that HPV positivity was significantly correlated with an increased risk of high-grade cervical lesions in AGC women, though the risk of high-grade lesions in women with AGC who were HPV-negative was also elevated 30. Notably, our study identified 18 hrHPV-negative cases with malignant pathological diagnoses, 50% of which were gastric ECA. This highlights the need for more effective additional tests to ensure accurate screening. Many studies have shown that gastric ECA usually has a shorter survival time and a high recurrence rate 31. CA19-9, a commonly-used tumor marker, has been widely considered a biomarker contributing to the diagnosis, treatment, and prognosis of gastric ECA 31.

In further stratified analysis of hrHPV subtypes and age in women with the AGC-NOS group, we found that hrHPV-positive status and age of 30 years or older were risk factors for predicting CIN3+ lesions, with HPV16-positive or 18/45-positive status and age between 40 and 49 years having the greatest impact on CIN3+ lesions. Similar conclusions were reached by Norman *et al.*, who found that HPV16/18-positive women had the highest cumulative incidence of CIN3+ lesions 32. A study by Kim *et al.* also supported this finding 33. In our study, the immediate risk of CIN3+ lesions in HPV-/AGC-NOS women younger than 30 years old was 0.0%, slightly lower than the 1.1% reported by the U.S. KPNC database 34. Since the immediate risk of CIN3+ in these cases is lower than the 4.0% threshold for immediate colposcopy, it is acceptable to defer the timing of colposcopy and opt for short-term follow-up, such as 1 year, based on a risk-based management strategy 15.

## Conclusion

Women with abnormal cervical glandular cytology often exhibit high-grade lesions. Apart from cervical malignancies, endometrial cancer, ovarian cancer, and fallopian tube cancer are also prevalent malignant tumors. Younger women (<30 years old) with AGC-NOS and HPV-negative status may be candidates for delaying colposcopy and extending the follow-up period to 1 year. This study includes a large number of Chinese women, which is what the previous studies lacked. For a developing country like China, this finding could significantly reduce the probability of referring to the colposcopy, which could reduce the burden of national medical insurance and the psychological burden of patients.

## Figures and Tables

**Table 1:** The immediate histologic results in women with abnormal cervical glandular cytology.

Cytologic diagnosis	Benign	CIN1	CIN2	CIN3	AIS	Cervical carcinoma			Non-cervical carcinoma			Total
						AC	SCC	Other types	Uterine	Ovary	GI tract	
AGC-NOS	202 (67.6%)	53 (17.7%)	6 (2.0%)	12 (4.0%)	13 (4.3%)	10 (3.3%)	2 (0.7%)	1 (0.3%)	0	0	0	299 (74.2%)
AGC-N	19 (27.1%)	8 (11.4%)	4 (5.7%)	10 (14.3%)	6 (8.6%)	13 (18.6%)	2 (2.9%)	1 (1.4%)	3 (4.3%)	1 (1.4%)	3 (4.3%)	70 (17.4%)
AIS	0	1 (11.1%)	0	1 (11.1%)	4 (44.4%)	2 (22.2%)	0	0	1 (11.1%)	0	0	9 (2.2%)
AC	1 (4.0%)	0	1 (4.0%)	0	0	18 (72.0%)	2 (8.0%)	1 (4.0%)	1 (4.0%)	1 (4.0%)	0	25 (6.2%)
**Total**	222 (55.1%)	62 (15.4%)	11 (2.7%)	23 (5.7%)	23 (5.7%)	43 (10.7%)	6 (1.5%)	3 (0.7%)	5 (1.2%)	2 (0.5%)	3 (0.7%)	403

**Table 2:** The HPV results in women with abnormal cervical glandular cytology.

Cytologic diagnosis	HPV16+	HPV18/45+	Other 11 hrHPV+	HPV16, 18/45+	hrHPV-	Total
AGC-NOS	19 (6.4%)	19 (6.4%)	41 (13.7%)	0	220 (73.6%)	299
AGC-N	18 (25.7%)	14 (20.0%)	15 (21.4%)	1 (1.4%)	22 (31.4%)	70
AIS	0	6 (66.7%)	0	0	3 (33.3%)	9
AC	8 (32.0%)	4 (16.0%)	2 (8.0%)	0	11 (44.0%)	25
**Total**	45 (11.2%)	43 (10.7%)	58 (14.4%)	1 (0.2%)	256 (63.5%)	403

**Table 3:** The immediate risk of cervical cancer and precancer in women with abnormal cervical glandular cytology.

Cytologic diagnosis	CIN3+			P-value
	n/N	Immediate risk (95%CI)	Relative risk (95%CI)	
AGC-NOS	38/299^*#@^	12.7% (9.26-17.15)	1.000	^*^P<0.001 ^#^P<0.001 ^@^P<0.001^&^P<0.001
AGC-N	39/70^*&^	55.7% (43.39-67.40)	2.0 (1.51-2.57)	
AIS	8/9^#^	88.9% (50.67-99.42)	7.9 (1.24-49.89)	
AC	23/25^@&^	92.0% (72.50-98.60)	10.9 (2.89-41.26)	
**Total**	108/403	26.8% (22.59-31.46)	NA	

**Table 4:** HPV genotype-stratified immediate histopathological correlation among women with abnormal cervical glandular cytology.

HPV genotype	CIN3+			P-value
	n/N	Immediate risk (95% CI)	Relative risk (95% CI)	
Negative	34/256^*#^	13.3% (9.49-18.20)	1.00	^*^P<0.001 ^#^P<0.001 ^@^P<0.001 ^&^P<0.001
16+	28/45^*@^	62.2% (46.54-75.84)	2.3 (1.57-3.35)	
18/45+	31/43^#&^	72.1% (56.09-84.16)	3.1 (1.92-5.04)	
Other 11 types+	15/58^@&^	25.9% (15.65-39.29)	1.2 (1.00-1.37)	
16 and 18/45+	0/1	0.0% (0.00-94.54)	NA	
**Total**	108/403	26.8% (22.59-31.46)	NA	

**Table 5:** Age-stratified immediate histopathological correlation among women with abnormal cervical glandular cytology.

Age group	CIN3+			P-value
	n/N	Immediate risk (95% CI)	Relative risk (95% CI)	
<30	7/53^*#^	13.2% (5.92-25.96)	1.0	^*^P=0.011 ^#^P=0.011
30-39	27/117	23.1% (16.01-31.96)	1.1 (0.98-1.30)	
40-49	41/130^*^	31.5% (23.83-40.36)	1.3 (1.08-1.48)	
≥50	33/103^#^	32.0% (23.38-42.06)	1.3 (1.08-1.51)	
**Total**	108/403	26.8% (22.59-31.46)	NA	

**Table 6:** Immediate risk assessment of AGC-NOS women based on age and HPV status.

HPV genotype	Age group				Total
	<30	30-39	40-49	≥50	
Negative	0/35 (0.0%)	2/64 (3.1%)	1/66 (1.5%)	9/55 (16.4%)	220
16+	0/1 (0.0%)	5/7 (71.4%)	3/6 (50.0%)	1/5 (20.0%)	19
18/45+	1/2 (50.0%)	2/5 (40.0%)	4/8 (50.0%)	1/4 (25.0%)	19
Other 11 types+	0/3 (0.0%)	5/23 (21.7%)	1/8 (12.5%)	3/7 (42.9%)	41
**Total**	1/41 (2.4%)	14/99 (14.1%)	9/88 (10.2%)	14/71 (19.7%)	299
